# Retroperitoneal bulk predicts chylothorax in follicular lymphoma–control requires systemic response

**DOI:** 10.1038/s41375-026-03025-x

**Published:** 2026-07-06

**Authors:** Denis Terziev, Franziska Lea Schümann, Timo Behlendorf, Franziska Brunner, Stephan Eisenmann, Elisabeth Groß, Michael Heuser, Thomas Weber

**Affiliations:** 1 Department of Internal Medicine IV, Hematology and Oncology, Universitätsmedizin Halle (Saale), University Hospital Halle (Saale), Martin-Luther-University Halle-Wittenberg, Halle (Saale), Germany; 2 Department of Internal Medicine I, Gastroenterology and Pulmology, Universitätsmedizin Halle (Saale), University Hospital Halle (Saale), Martin-Luther-University Halle-Wittenberg, Halle (Saale), Germany; 3 Onkologie MVZ III der Evidia MVZ Halle (Saale) GmbH, Halle (Saale), Germany

**Keywords:** Cancer therapy, Signs and symptoms, Risk factors

## To the Editor

Lymphoma-associated non-traumatic chylothorax (NTC) is defined as the accumulation of triglyceride-rich pleural fluid (>1.24 mmol/L) due to a lymphoproliferative disorder [1]. It may cause dyspnea, respiratory failure, increase the risk of infections such as pleural empyema, and exacerbate malnutrition [2]. Specific therapeutic options for NTC include conservative procedures such as thoracentesis or drain insertion, nutritional management involving total bowel rest with parenteral nutrition or a low-fat diet rich in medium-chain triglycerides (MCT) [2], as well as pharmacologic treatment with octreotide or somatostatin [3]. Additionally, various interventional strategies directed at the thoracic duct [4] or pleural space [5] have demonstrated efficacy. Although malignant lymphoproliferative disorders cause the majority of NTC cases, lymphoma-associated NTC is a rare complication and has mainly been described in sporadic case reports and series [4, 6, 7]. Because entity-specific features differ substantially between lymphoma subtypes, we focused this analysis exclusively on follicular lymphoma. In the absence of established guidelines or clinical trials, no standard of care exists for managing NTC in patients with FL and therapeutic decisions are highly individualized.

To systematically investigate the characteristics and management of NTC in follicular lymphoma (FL), we performed a retrospective observational analysis of six patients treated at our institution between 2017 and 2025 and complemented these cases with a PRISMA-guided literature review, yielding a total cohort of 31 FL-associated NTC cases (Fig. S1). The identified publications date from 1995 to 2024. Patient and disease characteristics are summarized in Supplementary Table S1 and compared with two previously published FL cohorts. Detailed methodology, including inclusion criteria, definitions, and ethical considerations, is provided in the Supplementary Appendix.

The analyzed patients had a median age of 62 years (range 35–78 years) at the time of NTC diagnosis, with a nearly equal sex distribution (male-to-female ratio 0.88). Twelve of 15 patients with available histological data were diagnosed with FL grade 1–2 according to the World Health Organization (WHO) classification (80%), and three patients had FL grade 3 A (20%). Patients with FL grade 3B or histological transformation were excluded, as they differ substantially from classical follicular lymphoma. Among the 24 patients with available Ann Arbor staging, one patient (4%) had stage II disease, three patients (13%) had stage III, and 20 patients (83%) had stage IV disease. Twenty-three out of 24 patients (85%) had nodal retroperitoneal FL manifestations. Eleven patients (46%) had additional nodal thoracic involvement, and one patient (4%) had isolated nodal thoracic involvement. Seventeen of 18 evaluable patients (94%) had a lymphoma bulk >7 cm. All 17 patients showed retroperitoneal bulk localization. Unilateral effusions (63%) were more common than bilateral effusions (37%), without a clear predominance of left- (*n* = 7, 37%) or right-sided (*n* = 5, 26%) involvement. Levels of reported triglycerides within the NTC were widely spread with a median of 9.4 mmol/L (range 1.8–79 mmol/L). Seven of 18 evaluable patients (39%) had concomitant chyloperitoneum at the time of NTC diagnosis. A detailed summary of all patient and disease characteristics is provided in Supplementary Table S2.

**Fig. 1:**
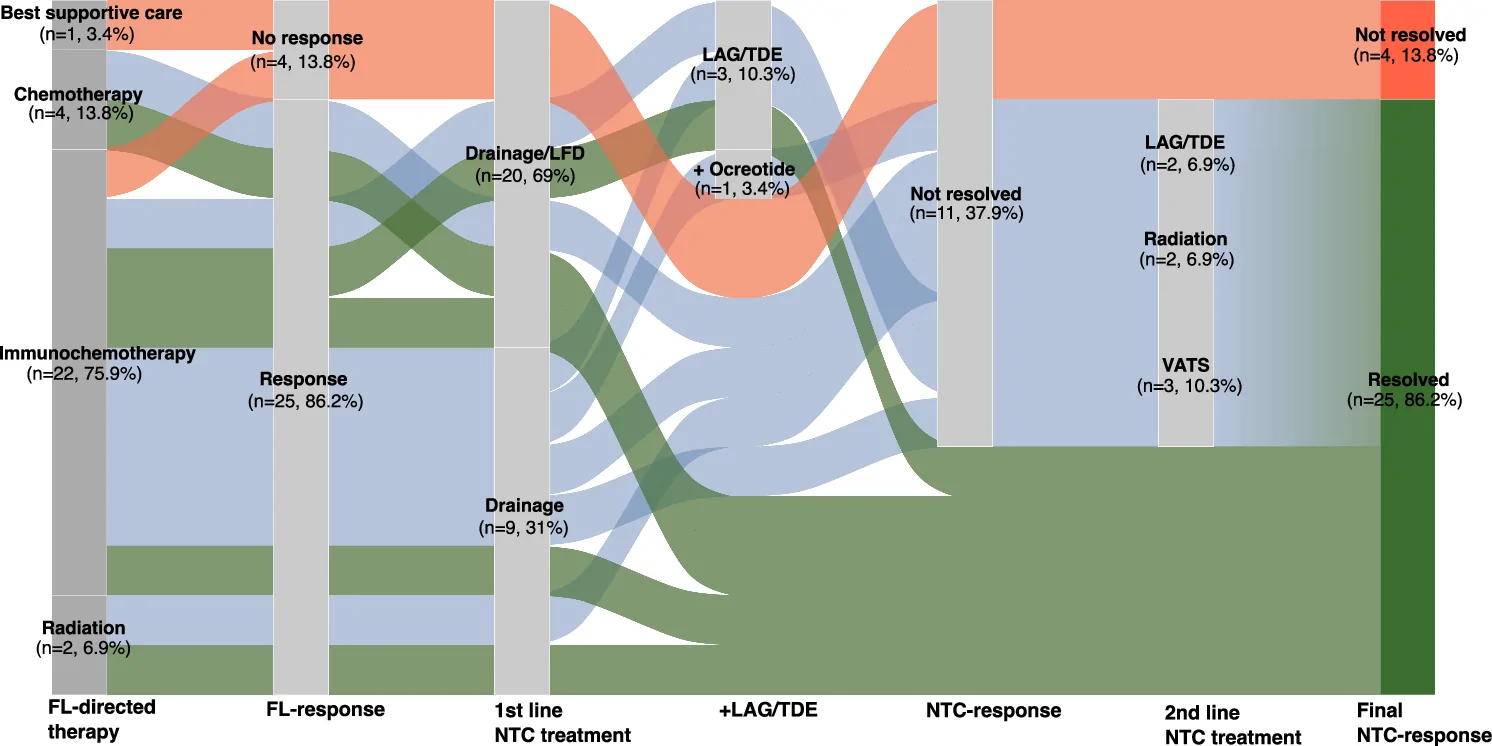
Treatment patterns and outcomes of FL and NTC of the total cohort with evaluable data (n = 31). Abbreviations: FL follicular lymphoma, LFD low-fat diet, LAG / TDE lymphangiography/ thoracic duct embolization, NTC non-traumatic chylothorax, VATS video-assisted thoracoscopy.

**Fig. 2:**
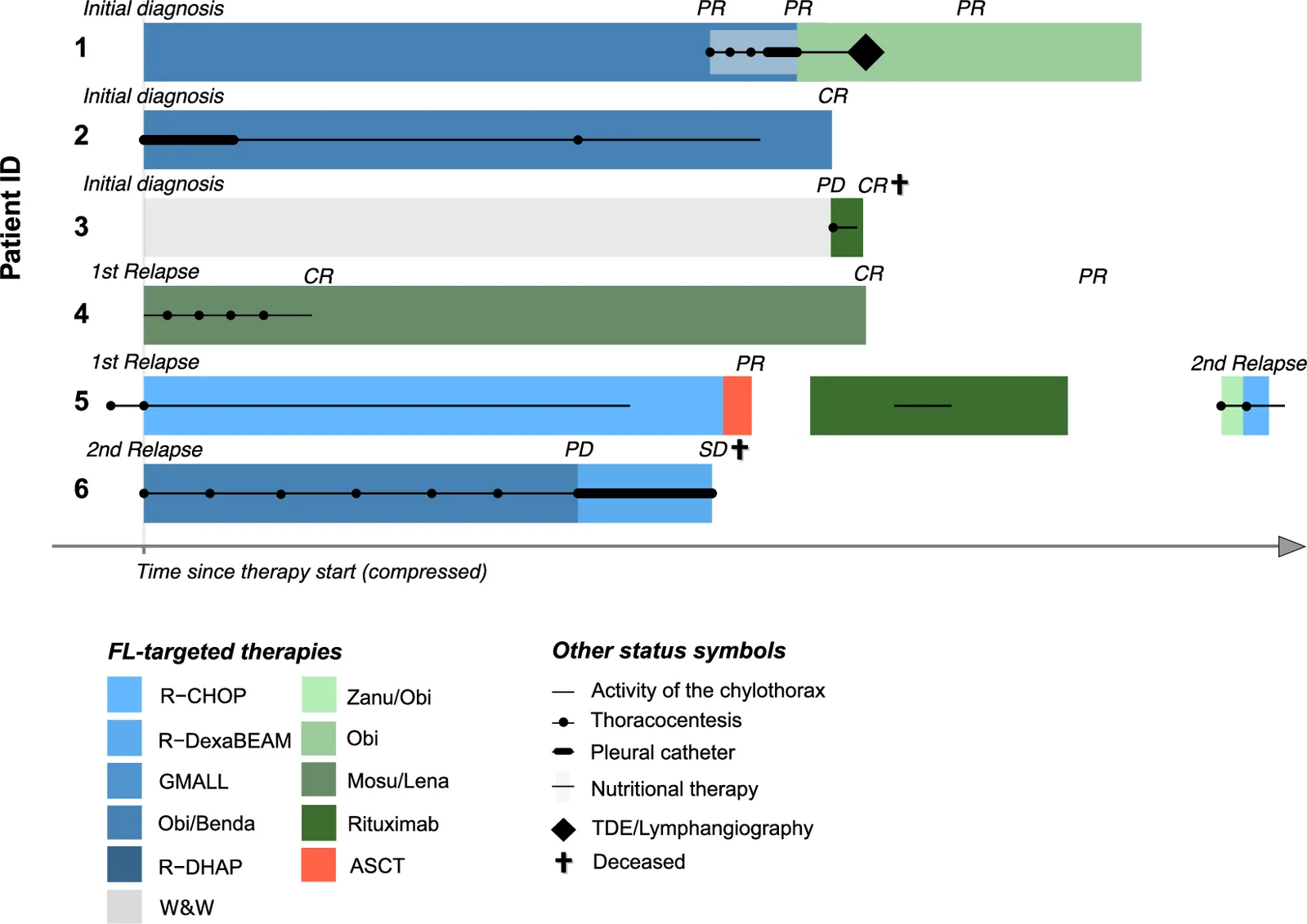
Swimmer plot illustrating the treatment courses and clinical outcomes of six patients with follicular lymphoma-associated non-traumatic chylothorax treated at the University Hospital Halle (Saale). Abbreviations: ASCT autologous stem cell transplantation, Benda bendamustine, CART chimeric antigen receptor T-cell therapy, CR complete response, GMALL therapy according to the protocol of the German Multicenter Study Group for Adult Acute Leukemia, Lena lenalidomide, Mosu mosunetuzumab, NTC non-traumatic chylothorax, PD progressive disease, PR partial response, ID identification number, R-CHOP rituximab, cyclophosphamide, doxorubicin, vincristine, and prednisone, R-DexaBEAM, rituximab, dexamethasone, carmustine, etoposide, cytarabine, melphalan, R-DHAP, rituximab, dexamethasone, cytarabine, and cisplatin, R-ICE, rituximab, ifosfamide, carboplatin, and etoposide, SD stable disease, TDE Thoracic duct embolization, VATS video-assisted thoracoscopic surgery, W&W watch and wait, Zanu zanubrutinib.

NTC was present in 26 of 31 evaluated patients (84%) at the time of initial FL diagnosis (first-line therapy). In four cases (13%), NTC occurred during second-line therapy, and in one case (3%), it appeared during third-line therapy. If reported, 28 of 29 evaluable cases received FL-directed therapy, which comprised (immuno-)chemotherapy (*n* = 26; 90%) and radiation (*n* = 2; 7%). One patient received best supportive care (4%). Out of 29 patients with reported data on therapy outcome, four patients (14%) did not respond to the FL-directed therapy. One patient (3%) showed FL response, yet died of pulmonary embolism shortly after therapy initiation. None of the patients with insufficient FL control showed NTC response despite conservative treatment (*n* = 4, 100%). It should be noted, however, that these patients only received paracentesis of the effusions, without any further NTC-directed therapy. Treatment patterns and outcomes of FL and NTC across all patients with available treatment courses and outcomes (*n* = 29) are summarized in Fig. 1. The individual treatment courses of the six institutional cases are depicted in Fig. 2, illustrating the temporal relationship between FL-directed therapy and NTC resolution. In the cohort of patients who did respond to FL-directed therapy and did not experience complications (*n* = 25; one fatal), eleven patients (44%) did not achieve remission of NTC with first-line NTC-directed therapy. Of these, seven patients subsequently received salvage NTC therapy. Salvage NTC therapy included interventional approaches such as radiation of the celiac and thoracic duct (*n* = 2), video-assisted thoracoscopic surgery (VATS) with pleurodesis (*n* = 3), thoracic duct embolization or ligation (*n* = 2). No meaningful difference in outcome was observed between right- and left-sided chylothorax (right: 6/6, 100%; left: 5/6, 83%). All patients received these measures alongside ongoing FL-directed therapy, resulting in complete NTC remission in all remaining cases. A detailed overview of therapy characteristics and clinical outcomes is provided in Supplementary Table S2.

To our knowledge, this is the largest report on clinical presentation and treatment-specific outcomes of FL-associated NTC, combining an institutional core series of six cases with a PRISMA-based literature review. Overall, we showed that retroperitoneal bulk was associated with NTC and that control of FL was indispensable for NTC management. However, additional NTC-specific treatments were necessary to achieve resolution of NTC in a significant number of cases. The compiled cases are comparable to other FL patients regarding age, sex, AA stage and FL grading [8–10]. Interestingly, lymphoma bulks > 7 cm were present in 94% of the NTC-affected patients in our analysis, which is notably higher than the prevalence reported in established FL cohorts. Previous studies have reported rates of 27% [11]. 44% [12]. and 66.3% [13]. respectively. A large majority of these patients had retroperitoneal bulky disease and not necessarily intrathoracic FL manifestations. Moreover, chylous effusions were not preferentially observed within the right pleural cavity as described for chylothoraces in general [14]. but occurred often bilaterally with or without concomitant chyloperitoneum. Consistently, no meaningful difference in outcome was observed between right- and left-sided chylothorax. Taken together, these observations indicate that FL-associated NTC might not exclusively result from damage and consecutive leakage of the predominantly right-sided thoracic duct but can also be a consequence of tumor-compression of lymphatic vessels resulting in extravasation of chyle into the body cavities [1, 15].

Since no patient without response to FL-directed treatment showed NTC remission, the current data support that FL control is indispensable for successful treatment of NTC [4, 6]. On the other hand, sufficient control of FL burden may not necessarily improve the control of the chylothorax. In these cases, additional NTC-directed therapy is needed. Due to a wide range of possible NTC-directed treatment options and a lack of comprehensive case series, it remains unclear which NTC treatment might be most effective. Regarding nutritional strategies, Pospiskova et al. [6] reported on the successful treatment of ten patients with refractory FL-associated NTC by sufficient FL-directed treatment and an additive low-fat diet with parenteral nutrition. This approach led to a mean chylothorax duration of 53 days (25–82 days). However, the diet-based approach may not be well tolerated by single patients [6] or prove ineffective [15]. In case of persisting symptomatic NTC despite adequate FL control and failure of nutritional management, interventional NTC therapies and medication with octreotide showed efficacy [4, 15].

Although limited by sample size and retrospective design, our findings provide the first quantitative evidence linking anatomical disease distribution and therapeutic response in FL-associated chylothorax. Therefore, salvage NTC treatment may be selected in view of the underlying pathophysiology of the NTC as it may be evaluated by lymphangiography, the patient’s preference and availability of therapies.

In summary, retroperitoneal bulky disease represents a major anatomical risk factor for FL-associated chylothorax. Sustained resolution requires systemic lymphoma control, with interventional strategies serving as essential adjuncts in one-third of the cases. Nutritional and pharmacological measures remain secondary due to their limited long-term efficacy.

## Supplementary information

Supplemental Material

Supplemental Figure 1

## Data Availability

The dataset generated and analyzed during the current study is available from the corresponding author upon reasonable request.
