# Supplementary material for: Retroperitoneal bulk predicts chylothorax in follicular lymphoma–control requires systemic response
Source: Leukemia. 2026 Jul 6;40(9):2039–42. doi: 10.1038/s41375-026-03025-x (PMC13506320; doi:10.1038/s41375-026-03025-x)
Supplement: Supplementary file 1 [file 41375_2026_3025_MOESM2_ESM.pdf]

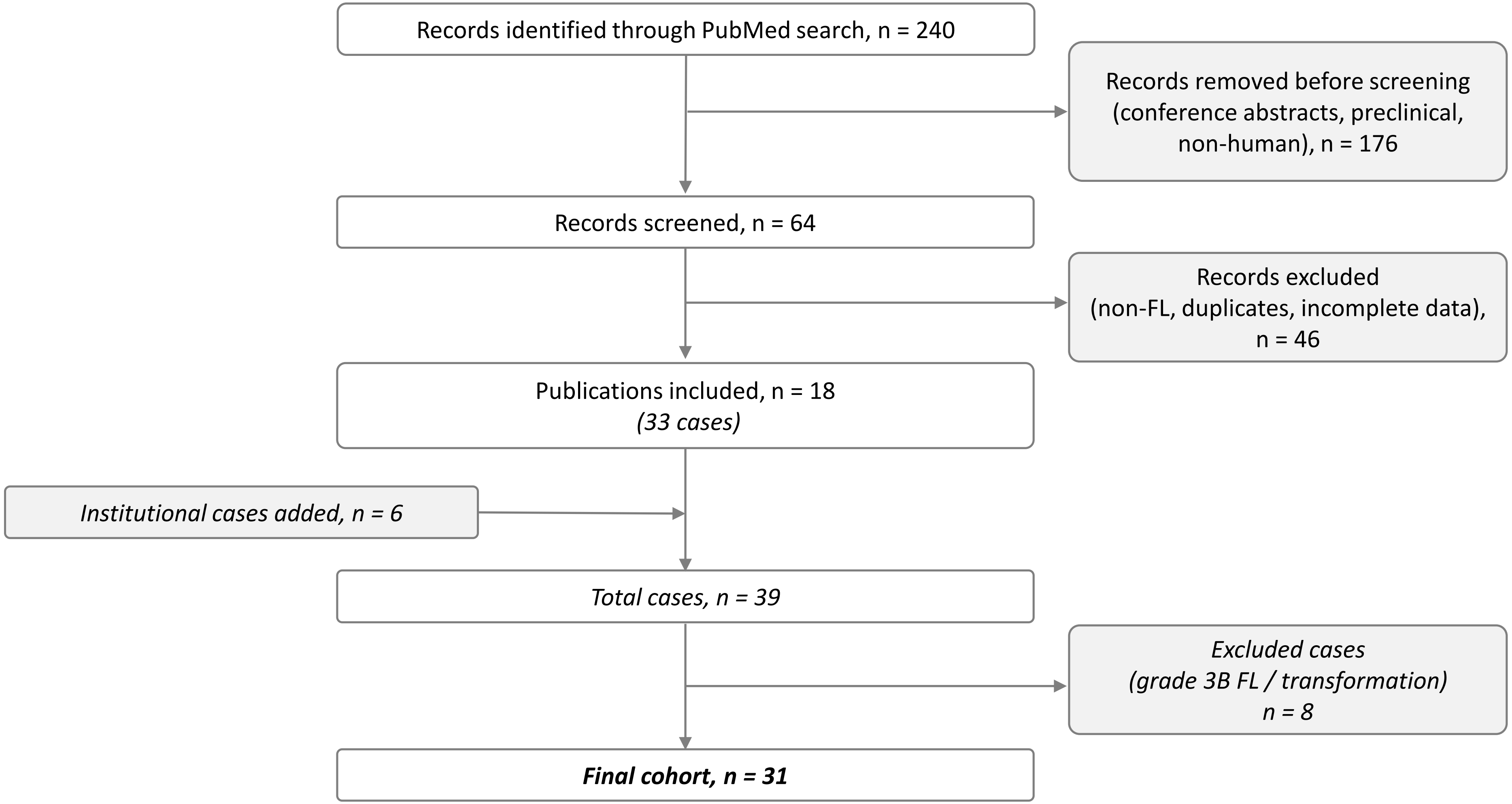
Records identified through PubMed search, n = 240
Records removed before screening (conference abstracts, preclinical, non-human), n = 176
Records screened, n = 64
Records excluded (non-FL, duplicates, incomplete data), n = 46
Publications included, n = 18
(33 cases)
Institutional cases added, n = 6
Total cases, n = 39
Excluded cases (grade 3B FL / transformation) n = 8
Final cohort, n = 31
