# Supplementary material for: Retroperitoneal bulk predicts chylothorax in follicular lymphoma–control requires systemic response
Source: Leukemia. 2026 Jul 6;40(9):2039–42. doi: 10.1038/s41375-026-03025-x (PMC13506320; doi:10.1038/s41375-026-03025-x)
Supplement: Supplementary file 2 [file 41375_2026_3025_MOESM1_ESM.docx]

**Supplementary Material**

**Supplementary Case Descriptions**

The clinical courses of the six patients with FL-associated NTC treated at the University Hospital Halle (Saale) between 2017 and May 2025 are described in detail below. The cases were presented in the order of chylothorax first manifestation within the FL history. *Figure 1* provides a graphical overview of the therapies administered and the corresponding clinical outcomes.

**Patient 1:** A 69-year-old man with follicular lymphoma (FL) (World Health Organization (WHO) grade 1-2, Ann Arbor stage (AA) IIIB), Follicular Lymphoma International Prognostic Index (FLIPI [1]) intermediate risk) presented with retroperitoneal bulky disease and bilateral nontraumatic chylothorax (NTC). Partial remission (PR) was achieved with obinutuzumab and bendamustine, but a right-sided NTC persisted despite additional nutritional measures (low-fat diet and medium-chain triglycerides (MCT), followed by parenteral nutrition and pleural catheter). Video-assisted thoracoscopic surgery (VATS) pleurodesis led to complete resolution of NTC, while FL remained in remission.

**Patient 2:** A 74-year-old woman with FL (WHO grade 1-2, AA IVEB, FLIPI 3), presenting with retroperitoneal bulky and right-sided NTC. Systemic therapy with obinutuzumab and bendamustine resulted in PR of lymphoma and complete resolution of the NTC without additional interventions.

**Patient 3:** An 80-year-old woman with previously observed FL, developed advanced-stage FL WHO grade 1-2 and retroperitoneal bulk presented with right-sided NTC (AA IIIA, FLIPI 3). Rituximab monotherapy resulted in prompt resolution of both lymphoma and NTC without additional interventions. The patient died shortly thereafter from an unrelated complication.

**Patient 4:** A 58-year-old man with FL after PR with obinutuzumab and cyclophosphamide, doxorubicin, vincristine, and prednisone (CHOP) presented with disease progression (WHO grade 1-2, AA IVA, FLIPI 3) with retroperitoneal bulky disease, ascites, and left-sided NTC. After 3 cycles of mosunetuzumab plus lenalidomide PET-CT demonstrated complete to partial metabolic remission with resolution of effusions. Twelve cycles were completed; two and a half years later, remission and NCT resolution persisted.

**Patient 5:** A 61-year-old woman with relapsed FL (WHO grade 1-2, AA IVA, FLIPI 3), presented with NTC with pleural involvement. Second-line R-CHOP followed by autologous stem cell transplantation (ASCT), resulted in PR and subclinical NTC residues. In February 2025, progressive FL (AA IIIB, FLIPI 4) and chylothorax were diagnosed. After unsuccessful obinutuzumab and zanubrutinib treatment, R-CHOP lead to stable disease with persistent effusion.

**Patient 6:** A 35-year-old man with relapsed FL (WHO grade 2-3A, AA IV, FLIPI 4) presented with retroperitoneal bulk, chylous ascites, and bilateral NTC. Disease was refractory to multiple systemic immunochemotherapies. Multiple paracenteses, thoracenteses, and pleural catheter insertion provided symptomatic relief. The patient died with persistent effusions but no resolution of NTC.

**Supplementary Methods**

**Study design and setting:** This study is a retrospective, exploratory, observational analysis of patients diagnosed with non-traumatic chylothorax (NTC) associated with follicular lymphoma (FL). Only patients with indolent FL (WHO grade 1–3A) were included. Cases with grade 3B FL or histological transformation were excluded. Institutional cases were identified through the hospital information system at University Hospital Halle (Saale), Germany, covering the period from January 2017 to May 2025.

**NTC definition and confirmation:** NTC was defined as an atraumatic accumulation of pleural fluid with triglyceride concentrations >1.24 mmol/L. Diagnosis was confirmed by chemical analysis of pleural fluid. Concomitant chyloperitoneum was recorded when present.

**Literature review:** A systematic PubMed search was conducted using the terms “lymphoma” AND “chylothorax,” following PRISMA guidelines. A total of 240 records were identified through PubMed search. After removal of conference abstracts, preclinical and non-human studies (n = 176), 64 records were screened. Of these, 18 publications met inclusion criteria, providing sufficient clinical information for analysis. These were combined with 6 institutional cases (total n = 39). After exclusion of 8 cases with grade 3B FL or histological transformation, the final cohort comprised 31 patients. A PRISMA flow diagram is provided in Supplementary Figure 1.

**Statistical analysis:** Descriptive statistics were performed using SPSS (version 29.0) and RStudio (version 4.3.3). Continuous variables are presented as median and range, categorical variables as number and percentage. Therapy response for FL and NTC was evaluated descriptively. No hypothesis testing was performed given the exploratory and retrospective nature of the study.

**Ethical considerations:** All data were analyzed retrospectively and anonymously in accordance with the Declaration of Helsinki and local data protection regulations (§15 of the professional code of the Medical Association of Saxony-Anhalt, Germany; reference number: 2019-072).

**Supplementary References**

1.	Solal-Celigny P. Follicular Lymphoma International Prognostic Index. Curr Treat Options Oncol. 2006;7(4):270-5. 10.1007/s11864-006-0036-3: {10.1007/s11864-006-0036-3}

2.	Nastoupil LJ MF, Scholz CW, Bishton MJ, Yoon SS, Giri P, et al. (2022) P1125: CELESTIMO: A Phase III Trial Evaluating the Efficacy and Safety of Mosunetuzumab Plus Lenalidomide Versus Rituximab Plus Lenalidomide in Patients With Relapsed or Refractory Follicular Lymphoma. Hemasphere. 6(Suppl):1015-1016. {10.1097/01.HS9.0000847368.92731}.

3.	Hiddemann W, Barbui AM, Canales MA, Cannell PK, Collins GP, Durig J, et al. Immunochemotherapy With Obinutuzumab or Rituximab for Previously Untreated Follicular Lymphoma in the GALLIUM Study: Influence of Chemotherapy on Efficacy and Safety. J Clin Oncol. 2018;36(23):2395-404. 10.1200/JCO.2017.76.8960: {10.1200/JCO.2017.76.8960}

4.	Barraclough A, England JT, Villa D, Wight J, Hapgood G, Conn J, et al. Outcomes in grade 3B follicular lymphoma: an international study led by the Australasian Lymphoma Alliance. Haematologica. 2023;108(9):2444-53. 10.3324/haematol.2022.281375: {10.3324/haematol.2022.281375}

5.	Ekeke CN, Chan EG, Luketich JD, Dhupar R. Delayed Chylothorax during Treatment of Follicular Lymphoma with a Malignant Pleural Effusion. Case Rep Surg. 2020;2020:2893942. 10.1155/2020/2893942: {10.1155/2020/2893942}

6.	O'Callaghan AM, Mead GM. Chylothorax in lymphoma: mechanisms and management. Ann Oncol. 1995;6(6):603-7. 10.1093/oxfordjournals.annonc.a059251: {10.1093/oxfordjournals.annonc.a059251}

7.	Van De Voorde L, Vanneste B, Borger J, Troost EG, Werner P. Rapid decline of follicular lymphoma-associated chylothorax after low dose radiotherapy to retroperitoneal lymphoma localization. Case Rep Hematol. 2014;2014:684689. 10.1155/2014/684689: {10.1155/2014/684689}

8.	Podder S, Mora M, Patel V, Sivamurthy S. A rare case of bilateral chylothorax: a diagnostic challenge--follicular lymphoma versus primary effusion lymphoma. BMJ Case Rep. 2015;201510.1136/bcr-2015-211935: {10.1136/bcr-2015-211935}

9.	Jagosky M, Taylor B, Taylor SP. A Case of Chyloperitoneum Secondary to Follicular Lymphoma and a Review of Prognostic Implications. Case Rep Hematol. 2016;2016:4625819. 10.1155/2016/4625819: {10.1155/2016/4625819}

10.	Gerstein J, Kofahl-Krause D, Fruhauf J, Bremer M. Complete remission of a lymphoma-associated chylothorax by radiotherapy of the celiac trunk and thoracic duct. Strahlenther Onkol. 2008;184(9):484-7. 10.1007/s00066-008-1840-4: {10.1007/s00066-008-1840-4}

11.	Mahajan A, Sankhyan P, Boonpheng B. Bilateral Chylothorax and Chylous Ascites: A Rare Presentation of an Uncommon Disorder. Cureus. 2021;13(3):e14044. 10.7759/cureus.14044: {10.7759/cureus.14044}

12.	Pospiskova J, Smolej L, Belada D, Simkovic M, Motyckova M, Sykorova A, et al. Experiences in the treatment of refractory chylothorax associated with lymphoproliferative disorders. Orphanet J Rare Dis. 2019;14(1):9. 10.1186/s13023-018-0991-3: {10.1186/s13023-018-0991-3}

13.	Teng CL, Li KW, Yu JT, Hsu SL, Wang RC, Hwang WL. Malignancy-associated chylothorax: a 20-year study of 18 patients from a single institution. Eur J Cancer Care (Engl). 2012;21(5):599-605. 10.1111/j.1365-2354.2012.01329.x: {10.1111/j.1365-2354.2012.01329.x}

14.	Fukumoto A, Terao T, Kuzume A, Tabata R, Tsushima T, Miura D, et al. Management of lymphoma-associated chylothorax by interventional radiology and chemotherapy: a report of five cases. Int J Hematol. 2022;116(4):579-85. 10.1007/s12185-022-03397-7: {10.1007/s12185-022-03397-7}

15.	Kim GE, Khaled Y, Mahmoud S, Ur Rehman A. (2024) Chylothorax as Initial Presentation of Follicular Lymphoma: A Case Report and Literature Search. Case Reports in Hematology.{10.1155/2024/7985228}.

16.	Krumina A, Auzina D, Legzdina A, Lejniece S. Chylothorax: A Tangled Road to Definitive Diagnosis of Non-Hodgkin Lymphoma. Am J Case Rep. 2023;24:e939098. 10.12659/AJCR.939098: {10.12659/AJCR.939098}

**Supplementary Tables**

**Table S1**

| **Characteristic** | **Current cohort** | **GALLIUM cohort[3]** |
| --- | --- | --- |
| **Age in years**, median (range) | 62 (35–78) | 59 (23–88) |
| **Sex, N (%)** |  |  |
| Female | 16 (52) | 638 (53) |
| Male | 15 (48) | 563 (47) |
| **FL WHO grade, N (%)** |  |  |
| 1–3a | 15 (100) | 1202 (100) |
| Unknown | 16 | — |
| **Ann Arbor stage, N (%)** |  |  |
| I–II | 1 (4) | 103 (9) |
| III–IV | 23 (96) | 1092 (91) |
| Unknown | 7 | — |
| **Bulky disease ≥ 7 cm, N (%)** |  |  |
| Yes | 17 (94) | 526 (44) |
| No | 1 (6) | 674 (56) |
| Unknown | 13 | 2 |

*Supplementary Table 1.* Patient and disease characteristics. Abbreviations: FL: follicular lymphoma; NTC: non-traumatic chylothorax.

**Table S2**

| **Patient**  **(publication year)** | **FL site** | **Bulk**  **(tumor >7 cm)** | **NTC site** | **Concomitant**  **Chyloperitoneum** | **FL therapy line** | **FL-directed therapy at time of NTC** | **FL response (at least remission)** | **1st-line NTC therape** | **NTC response to FL-directed therapy and 1st-line NTC therapy** | **Salvage NTC therapy** | **Final NTC response** |
| --- | --- | --- | --- | --- | --- | --- | --- | --- | --- | --- | --- |
| patient 1 | ret | ret | right | no | 1st | Obi Benda | response | LFD-MCT, PN, PC | not resolved | VATS-PD | resolved |
| patient 2 | ret | ret | left | no | 1st | Obi Benda | response | PC, TC | resolved | none | resolved |
| patient 3 | ret + tho | ret | left | yes | 1st | Rituximab | response | TC | resolved | none | resolved |
| patient 4 | ret + tho | ret | bilateral | yes | 2nd | Mosunetuzumab/ Lenalidomide | response | TC | resolved | none | resolved |
| patient 5 | ret + tho | ret | left | no | 2nd | R Benda, R-CHOP, ASCT, Zanubrutinib  /Obinutuzumab | no response | TC | not resolved | none | not resolved |
| patient 6 | ret + tho | ret | bilateral | yes | 3rd | Obi Benda, R-DexaBEAM | no response | PN, PC | not resolved | none | not resolved |
| patient 7 [5](2020) | ret + tho | ret | left | no | 1st | R Benda | response | LFD, PC, octreotide | not resolved | LAG/TDE | resolved |
| patient 8 [6] (1995) | ret | ret | bilateral | no | 1st | Chlorambucil Prednisolone | response | TC | not resolved | ligation | resolved |
| patient 9 [6] (1995) | ret | ret | bilateral | yes | 1st | PACEBOM, Chlorambucil Prednisolone | response | TC | resolved | none | resolved |
| patient 10 [7] (2014) | ret | ret | right | no | 1st | RTx bulk for  NTC 2nd line | response | TC, LFD-MCT | not resolved | Rx Bulk | resolved |
| patient 11 [8] (2015) | ret | no | bilateral | no | 1st | CHOP | n.m. | n.m. | n.m. | n.m. | n.m. |
| patient 12 [9] (2016) | ret | ret | left | yes | 1st | R-Benda | response | TC | resolved | none | resolved |
| patient 13 [10] (2008) | ret | ret | bilateral | no | 2nd | R-Benda | response | LFD-MCT | not resolved | Rx celiac + thoracic duct | resolved |
| patient 14 [11] (2021) | ret | ret | bilateral | yes | 1st | n.m. | n.m. | n.m. | n.m. | n.m. | n.m. |
| patient 15 [12](2019) | ret + tho | n.m. | n.m. | n.m. | 1st | Obi-Benda | response | PN | resolved | none | resolved |
| patient 16 [12](2019) | ret + tho | n.m. | n.m. | n.m. | 1st | Obi-Benda | response | TC, LFD, PN | resolved | none | resolved |
| patient 17 [12] (2019) | tho | n.m. | n.m. | n.m. | 2nd | FCR + RTx Bulk | response | TC, LFD, PN, Rx | resolved | none | resolved |
| patient 18 [12] (2019) | ret + tho | n.m. | n.m. | n.m. | 1st | R-CHOP | response | TC, LFD, PN | resolved | none | resolved |
| patient 19 [12] (2019) | ret + tho | n.m. | n.m. | n.m. | 1st | R-CHOP, ASCT | response | TC, LFD, PN | resolved | none | resolved |
| patient 20 [13] (2012) | n.m. | n.m. | n.m. | n.m. | 1st | CHOP | response | TC | resolved | none | resolved |
| patient 21 [13] (2012) | n.m. | n.m. | n.m. | n.m. | 1st | CVP | response | TC | resolved | none | resolved |
| patient 22 [13] (2012) | n.m. | n.m. | n.m. | n.m. | 1st | Best  supportive  care | no response | TC | not resolved | none | not resolved |
| patient 23 [13] (2012) | n.m. | n.m. | n.m. | n.m. | 1st | R-CHOP | no response | TC | not resolved | none | not resolved |
| patient 24 [13] (2012) | n.m. | n.m. | n.m. | n.m. | 1st | R-CHOP | response | TC | resolved | none | resolved |
| patient 25 [13] (2012) | n.m. | n.m. | n.m. | n.m. | 1st | R-CHOP | response | TC | resolved | none | resolved |
| patient 26 [13]  (2012) | n.m. | n.m. | n.m. | n.m. | 1st | R-CHOP | response | TC | resolved | none | resolved |
| patient 27 [14] (2022) | ret | ret | right | no | 1st | Obi-Benda | response | TC, LAG/TDE | resolved | none | resolved |
| patient 28 [14] (2022) | ret | ret | right | yes | 1st | R-CHOP, Obi-Benda | response | PC, PN, LAG/TDE | not resolved | VATS, pleurodesis | resolved |
| patient 29 [14] (2022) | ret | ret | bilateral | no | 1st | R-CHOP, ESHAP | response | octreotide, LAG/TDE | resolved | none | resolved |
| patient 30 [15](2024) | ret | ret | left | no | 1st | R-CHOP | response | TC | resolved | none | resolved |
| patient 31 [16](2023) | ret + tho | n.m. | right | no | 1st | R-CVP | response | VATS-PD, PN | resolved | none | resolved |

*Supplementary Table S2.* Disease, treatment, and outcome characteristics. Abbreviations: abd: abdominal, FL: follicular lymphoma, LAG/TDE: lymphangiography/thoracic duct embolization, LFD: low-fat diet, MCT: medium-chain triglycerides, n.m.: not mentioned, NTC: non-traumatic chylothorax, PC: pleural catheter, PN: parenteral nutrition, ret: retroperitoneal, RTx: radiation, TC: thoracocentesis, tho: thoracic, VATS-PD: video-assisted thoracoscopy–pleurodesis.

**Supplementary Figure Legends**

*Supplementary Figure S1.* PRISMA flow diagram of the systematic literature review. Records were identified through PubMed search (“lymphoma” AND “chylothorax”) between 1990 and 2025 (n = 240). After removal of conference abstracts, duplicates, non-English or non-FL, and preclinical and non-human studies (n = 176), 64 records were screened. Of these, 18 publications met inclusion criteria and contributed 33 literature cases. After addition of 6 institutional cases and exclusion of 8 cases with grade 3B FL or histological transformation, 31 cases were included in the final analysis. Abbreviations: FL, follicular lymphoma; PRISMA, Preferred Reporting Items for Systematic Reviews and Meta-Analyses.

**Supplementary Table Legends**

*Supplementary Table 1.* Patient and disease characteristics. Abbreviations: FL: follicular lymphoma; NTC: non-traumatic chylothorax.

*Supplementary Table S2.* Disease, treatment, and outcome characteristics. Abbreviations: abd: abdominal, FL: follicular lymphoma, LAG/TDE: lymphangiography/thoracic duct embolization, LFD: low-fat diet, MCT: medium-chain triglycerides, n.m.: not mentioned, NTC: non-traumatic chylothorax, PC: pleural catheter, PN: parenteral nutrition, ret: retroperitoneal, RTx: radiation, TC: thoracocentesis, tho: thoracic, VATS-PD: video-assisted thoracoscopy–pleurodesis.
